# Consensus-based framework of actions within educational and family settings to promote healthy and safe ICT use among adolescents: a Delphi study

**DOI:** 10.3389/fpubh.2026.1813279

**Published:** 2026-06-24

**Authors:** Andrés Arana-Rodríguez, Almudena Garrido-Fernández, Miriam Sánchez-Alcón, Julia Sánchez-Galloso, Francisco-Javier Gago-Valiente, Francisca María García-Padilla

**Affiliations:** 1 Department of Nursing, University of Huelva, Huelva, Spain; 2 Center for Research in Contemporary Thought and Innovation for Social Development (COIDESO), University of Huelva, Huelva, Spain

**Keywords:** adolescent health, community nursing, Delphi study, digital health, health promotion, public health

## Abstract

**Introduction:**

Adolescents are increasingly exposed to a rapidly evolving digital ecosystem that offers opportunities for learning and health promotion, but also poses risks such as problematic use, health-related misinformation, cyberbullying, and exposure to inappropriate content. Despite growing public concern and regulatory attention, gaps remain in clear, evidence-based guidance to support adolescents, families, schools, and other stakeholders in promoting healthy and safe use of information and communication technologies. The aim of this study was to establish expert consensus priorities to inform policy and public health practice.

**Material and methods:**

Between November 2024 and June 2025, a three-round Delphi study was conducted with a multidisciplinary panel of experts. In round 1, experts proposed measures in response to an open-ended question. Responses were organized into categories using researcher triangulation. In round 2, the experts indicated their agreement or disagreement with each measure. In the third round, importance was rated on a 10-point scale (1-10). Consensus was predefined as ≥80% agreement, and very high agreement as ≥95%. A composite relevance variable integrating agreement and importance was used to classify measures into three relevance levels.

**Results:**

Of 100 experts invited, 38 agreed to participate. The number of participants who responded in each round was 28 (Round 1), 22 (Round 2) and 17 (Round 3). The experts generated 184 actions grouped into the categories of educational sphere (*n* =108; 58.70%), family sphere (*n* = 60; 32.60%) and general actions (*n* = 16; 8.70%). In round 2, 134 actions (72.83%) reached the consensus threshold and 52 (28.26%) reached a very high agreement (≥95%). In the third round, 54 actions (29.35%) were classified in the highest importance range (9.0–10.0). Overall, 38 actions (20.65%) were classified as highly relevant, with most high-relevance actions related to families and educational centers.

**Conclusions:**

This study translates multidisciplinary expertise into a priority multisectoral action framework that emphasizes education and active mediation over predominantly restrictive approaches. The findings provide a basis for the design and implementation of coordinated measures across families, schools, and the community.

## 1 Introduction

According to the World Health Organization (WHO), adolescence is defined as the period between 10 and 19 years of age, during which substantial physical, psychological, and social changes take place (1). During this stage, the adoption of healthy habits is essential not only because of their immediate benefits, but also because of their consolidation and maintenance into adulthood, with potential implications for future generations (2).

Adolescent health is currently at a turning point, characterized by the emergence of new challenges and opportunities, particularly within digital and technological environments. In this regard, the second Lancet Commission on Adolescent Health and Wellbeing (3) highlights the increasingly central role that digital technologies play in adolescents' daily lives, shaping their emotional and social learning experiences through the internet. This process, described as the digital transition, offers new opportunities for personal development, including improved access to health promotion resources and educational platforms. At the same time, this transition introduces new risks associated with digital and online environments, such as health-related misinformation, social media algorithms that may encourage problematic use, cyberbullying, and exposure to inappropriate content.

Within this framework, the Committee on the Rights of the Child (4) emphasizes that technology must adhere to ethical and safety standards, preventing exposure not only to violence and discrimination, but also to the misuse of personal data. The materialization of these risks may compromise adolescent health. For example, internet addiction has been significantly associated with negative psychosocial outcomes and clinical symptoms (5). Similarly, higher levels of social media addiction have been linked to an increased likelihood of psychosocial difficulties (6). These concerns are consistent with a WHO report on adolescence (7), which documents a marked increase in problematic social media use between 2018 and 2022. In response to this trend, the report stresses the importance of adolescents achieving a healthy balance between online activities and offline pursuits, such as rest, physical activity, academic responsibilities, and social relationships with peers.

Alongside these issues, youth violence has also evolved in parallel with technological innovation, positioning cyberbullying as a significant and increasingly prevalent form of violence among adolescents. Consequently, it has become a major social concern, underscoring the urgent need to implement educational actions targeting young people, families, and educational institutions, as well as to establish regulatory frameworks for social media platforms that limit exposure to cyberbullying (8).

In this rapidly evolving digital ecosystem, marked by the progressive incorporation of new technologies, including artificial intelligence-based tools, growing attention has been paid to how opportunities and risks can be balanced, and to how adolescents can be adequately supported. In this context, one of the goals of the second Lancet Commission on Adolescent Health and Wellbeing (3) is to promote healthy use of social media and online environments, while preventing associated risks through a multisectoral and multilevel approach encompassing educational and family settings, adolescent participation, and collaboration between governmental institutions and other relevant stakeholders (3). Similarly, the Committee on the Rights of the Child (4) advocates for the use of digital technologies as a strategy for promoting healthy lifestyles, provided that appropriate safeguards are in place.

These issues have also received growing attention in Spain, where national legislation has progressively addressed the protection and digital education of minors. Organic Law 3/2018 on Personal Data Protection and Guarantee of Digital Rights promotes the responsible, critical, and safe use of digital media within the education system, including attention to risks associated with ICT use and online violence (9). In addition, Organic Law 8/2021 on the comprehensive protection of children and adolescents against violence highlights the importance of educational and awareness-raising actions aimed at promoting the safe and responsible use of the Internet among children, adolescents, families, educators, and other professionals (10). These concerns are also reflected in recent epidemiological studies conducted in representative samples of Spanish adolescents, which have drawn attention to problematic Internet use and problematic gaming among adolescents (11).

Beyond individual and technological factors, previous research highlights the importance of contextual influences. Vanden Abeele (12) argues that, to better understand and enhance the effectiveness of interventions aimed at digital wellbeing, it is necessary to broaden the focus to include contextual elements, acting both at the group level, such as through family rules, and at the institutional level. From this perspective, both the family and the educational center emerge as key settings for action.

Despite broad consensus that adolescents constitute a population particularly vulnerable to risks associated with digital environments, gaps remain in comprehensive, evidence-based recommendations to guide young people, their families, schools, and other stakeholders toward technology use that appropriately balances benefits and risks. In a recent report, the World Health Organization (13) identifies this lack of clear guidance as a strategic priority, noting that it hinders the translation of existing knowledge into actions applicable to everyday contexts.

Expert consensus can support the development of prioritized, actionable recommendations by integrating multidisciplinary perspectives, providing a starting point for subsequent stakeholder evaluation prior to implementation. Against this background, the present study aims to use the Delphi technique to identify and reach consensus on actions designed to promote the healthy and safe use of information and communication technologies (ICT) among adolescents, providing a structured and prioritized basis to inform practice.

## 2 Materials and methods

### 2.1 Study design

A Delphi study was conducted with the aim of reaching expert consensus on actions to promote the healthy and safe use of information and communication technologies among adolescents (14). The Delphi technique offers several advantages, including the ability to generate anonymous consensus among experts who may be geographically dispersed, through successive rounds of correspondence conducted by email. In addition, this method facilitates the expression of a wide range of perspectives while minimizing potential biases associated with face-to-face group dynamics (15). This Delphi study followed established reporting guidance for Delphi studies (CREDES) (16).

### 2.2 Participants

Selection criteria included professional experience in related fields (education, health, cybersecurity, and related areas), as well as scientific output, defined as authorship of books, scientific articles, doctoral theses, or other academic documents related to the study topic. A multidisciplinary panel was intentionally sought in order to incorporate health-related, socio-educational, and technical perspectives.

Initially, 100 individuals were invited to participate in the study, of whom 38 expressed interest and met the inclusion criteria. Of these, 28 participated in Round 1, 22 in Round 2, and 17 in Round 3. The composition of the expert panel across the Delphi rounds is presented in Table 1.

**Table 1:** Composition of the expert panel across the Delphi rounds.

Expert category	Round 1 *n* (%)	Round 2 *n* (%)	Round 3 *n* (%)
Secondary and vocational education teachers	14 (50%)	9 (40.91%)	9 (52.94%)
University lecturers/researchers	8 (28.57%)	7 (31.82%)	3 (17.65%)
Educational counselors	2 (7.14%)	2 (9.09%)	1 (5.88%)
Clinical psychologists	2 (7.14%)	2 (9.09%)	2 (11.76%)
Cybersecurity experts	2 (7.14%)	2 (9.09%)	2 (11.76%)
Total	28 (100%)	22 (100%)	17 (100%)

The final panel comprised cybersecurity experts, clinical psychology professionals, teaching staff with educational and guidance roles at secondary and upper secondary education and vocational training levels (predominantly in active service), and university teaching and research staff working in fields related to the topic of study. Secondary and vocational education teachers represented the largest professional group within the panel, followed by university lecturers/researchers. Sociodemographic characteristics of the participants in Round 1 are presented in Supplementary Table S1.

Most participants were based in Spain, predominantly in Andalusia, although the panel also included experts from other geographical contexts, which contributed to the diversity of perspectives represented in the study.

All participants provided informed consent to participate voluntarily. Confidentiality was guaranteed, and ethical research principles were observed throughout the study. Ethical approval was granted by the Huelva Provincial Research Ethics Committee.

### 2.3 Procedure

Once the Delphi panel was established, three questionnaire rounds were conducted between November 2024 and June 2025, with the aim of reaching consensus on actions for the healthy and safe use of ICT among adolescents.

Prior to the first round, the operational definition and the initial open-ended question were piloted with professionals who were not part of the final panel, in order to assess clarity and comprehensibility. Minor wording adjustments were made based on this pilot testing.

In the first round, participants were provided with:

1. An operational definition of safe ICT use was provided to participants, integrating both the safety dimension (protection against digital risks) and the health dimension (balanced use and health promotion). This ensured that experts shared a common conceptual framework encompassing both risk prevention and the promotion of wellbeing in digital environments.

2. An open-ended question inviting participants to propose actions within the educational and family settings aimed at promoting healthy and safe ICT use among adolescents.

A three-round design was adopted to progressively generate, agree upon, and prioritize actions. Based on responses from the first round, a content framework was developed and subsequently analyzed and structured through researcher triangulation. In the second round, participants were asked to indicate their agreement or disagreement with each proposed action. In the third round, participants rated the importance of each action using a 10-point scale (1 = lowest importance; 10 = highest importance). The questionnaire was developed for this study and is provided in English as Supplementary material S2.

Controlled feedback was provided between rounds. During the second round, participants received the full list of actions, while in the third round, information on the level of consensus reached for each action was included in order to encourage informed reflection.

### 2.4 Data analysis

To quantify the level of consensus, the percentage of agreement was calculated for each action based on responses to a dichotomous question assessing agreement or disagreement. The resulting percentages were subsequently grouped by category, subcategory, and thematic subsection, and mean agreement levels were calculated at each analytical level. A consensus threshold of 80% agreement was established. Actions reaching agreement levels of 95% or higher were considered to reflect a high level of consensus, allowing identification of the most strongly endorsed actions (17, 18).

For each action, the mean level of importance and standard deviation were calculated. The mean score was interpreted as an indicator of the overall importance assigned by the panel, while the standard deviation reflected the variability of responses among participants (19). Mean levels of importance were then grouped by category, subcategory, and thematic subsection, such that the resulting means represented the overall importance attributed at each level, and the corresponding standard deviations reflected variability across actions within each grouping.

To facilitate the synthesis and operational interpretation of the findings, actions were further classified according to both their level of agreement and level of importance. A composite variable termed *level of relevance* was defined. Actions were classified as having high relevance (Level I) when both criteria were high (percentage of agreement ≥95% and mean level of importance ≥9). Actions were classified as having moderate relevance (Level II) when agreement ranged between 80% and 94% and mean levels of importance ranged from ≥7 to < 9, or when one criterion met Level I thresholds while the other fell within the Level II range. Actions were considered to have limited relevance (Level III) when agreement was below 80% or mean levels of importance were below 7.

## 3 . Results

### 3.1 Generation of actions

**Figure 1:**
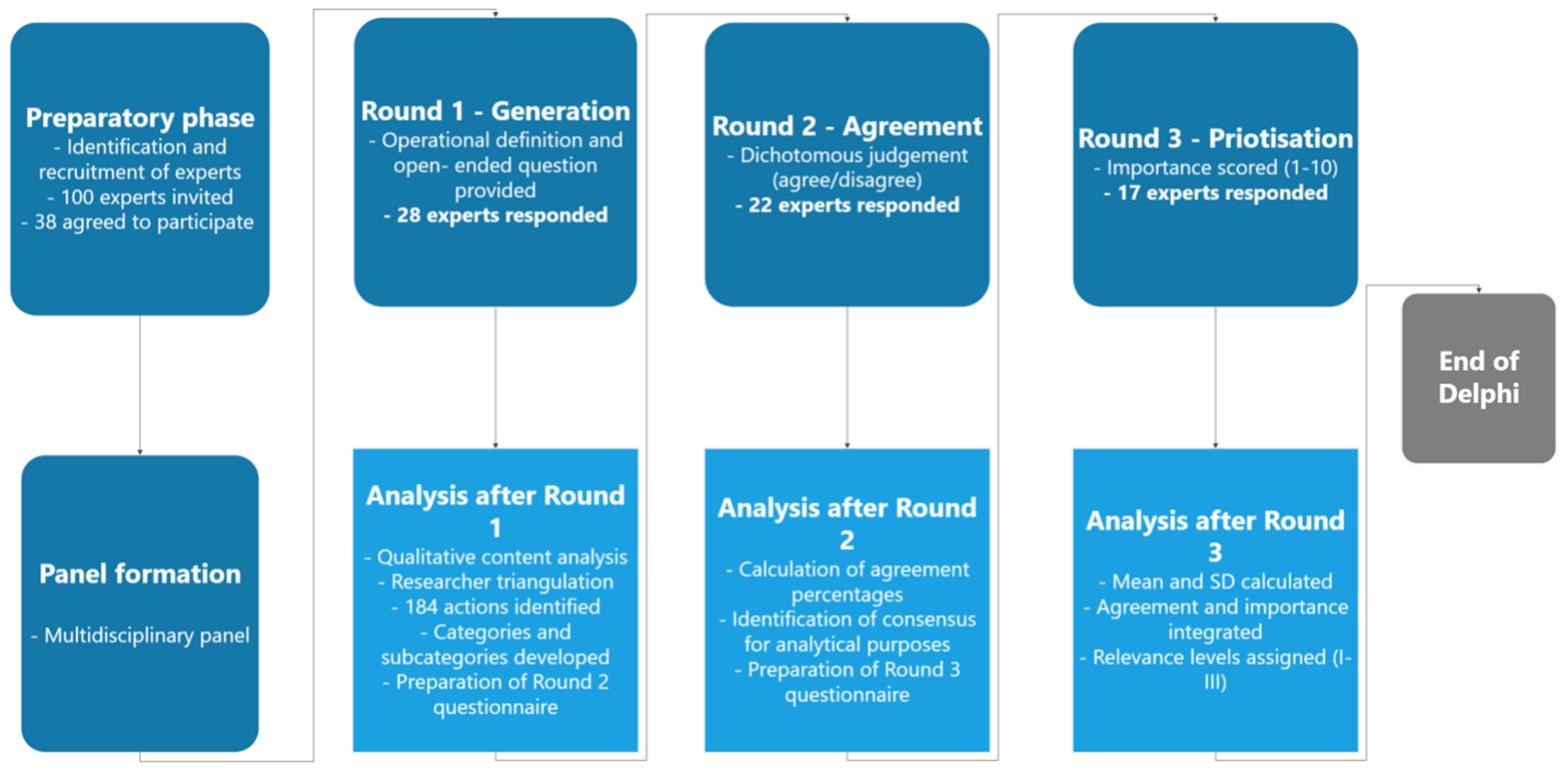
Flow chart of the Delphi process.

The aim of the first Delphi round was to generate an initial set of actions proposed by the expert panel, based on participants' open-ended contributions. Responses were received from 28 experts, thereby preserving the multidisciplinary composition of the panel across subsequent rounds (Figure 1).

Analysis of the responses enabled the organization of the information into categories, subcategories, and thematic subsections. A total of 184 actions were identified and grouped into three main categories: actions in the educational sphere (*n* = 108; 58.70%), actions in the family sphere (*n* = 60; 32.60%), and general actions (*n* = 16; 8.70%) (Table 2). Supplementary Table S3 provides illustrative examples derived from the proposed actions for each subcategory.

**Table 2:** Distribution of actions by category, subcategory, and thematic subsection.

Category	Subcategory	Thematic subsection	Number of actions	Percentage of total actions
Actions in the educational sphere	*Actions involving the educational center*	Creation and implementation of protocols and regulations for ICT device security and use	29	15.76%
		Resource mobilization and development of digital literacy programmes	8	4.35%
		Actions involving families and other specialists	13	7.07%
		Other actions	9	4.89%
	*Actions involving teachers*	Teacher training	3	1.63%
		Classroom-based actions	27	14.67%
	*Digital literacy and acquisition of psychosocial skills in students*		19	10.33%
Actions in the family sphere	*Actions involving parents/legal guardians/family*	Devices and their use within the family	20	10.87%
		Training and information for parents/legal guardians/families	6	3.26%
		Education and communication	20	10.87%
		Other actions	10	5.43%
	*Actions involving adolescents*		4	2.17%
General actions	*Policy and legislation*		9	4.89%
	*Science*		2	1.09%
	*Other actions*		5	2.72%
*Total actions*			184	100%

These three main categories were further subdivided into eight subcategories, three of which were organized into a total of ten thematic subsections (Table 2). The largest proportion of actions corresponded to the educational sphere (58.70%), with the thematic subsection ‘Creation and implementation of protocols and regulations for ICT device security and use' accounting for the highest percentage (15.76%). Actions in the family sphere represented 32.60% of the total, with greater concentration in the thematic subsections ‘Devices and their use within the family' (10.87%) and ‘Education and communication' (10.87%). General actions accounted for the remaining 8.70%, with ‘Policy and legislation' being the subcategory comprising the highest number of actions (4.89%).

### 3.2 Level of agreement

The second Delphi round focused on assessing the level of agreement for each action identified in the first round. A total of 22 experts participated in this round, representing a retention rate of 78.57% relative to the initial panel.

Participants were asked to indicate their agreement or disagreement with each action. As shown in Table 3, 81.52% of the actions achieved agreement levels between 76% and 100%, 13.59% fell within the 51–75% range, and only 4.89% obtained agreement levels below 50%.

**Table 3:** Distribution of agreement levels across action categories.

Agreement level interval	Total number of actions	Percentage of total actions	Number of actions by main category
0%−25%	3	1.63%	Educational sphere (*n* = 1)
			Family sphere (*n =* 2)
			General actions (*n =* 0)
26%−50%	6	3.26%	Educational sphere (*n =* 5)
			Family sphere (*n =* 1)
			General actions (*n =* 0)
51%−75%	25	13.59%	Educational sphere (*n =* 15)
			Family sphere (*n =* 8)
			General actions (*n =* 2)
76%−100%	150	81.52%	Educational sphere (*n =* 85)
			Family sphere (*n =* 51)
			General actions (*n =* 14)

Overall, 52 actions (28.26%) reached a very high level of agreement, defined as agreement equal to or greater than 95% (Table 4). Of these, 27 actions corresponded to the educational sphere, 20 to the family sphere, and 5 to general actions, representing 25.00%, 33.33%, and 31.25% of the actions within each category, respectively. In addition, 134 actions (72.83%) met the consensus threshold of 80% agreement or higher (Table 4). Mean agreement levels for each thematic subsection are presented in the Supplementary Table S4.

**Table 4:** Distribution of actions with agreement levels ≥80% and p30mm≥95%.

Category	Subcategory	Thematic subsection	Actions ≥80%	Actions ≥95%
Actions in the educational sphere	*Actions involving the educational center*	Creation and implementation of protocols and regulations for ICT device security and use	16	3
		Resource mobilization and development of digital literacy programmes	7	6
		Actions involving families and other specialists	11	4
		Other actions	4	0
	*Actions involving teachers*	Teacher training	3	0
		Classroom-based actions	19	9
	*Digital literacy and acquisition of psychosocial skills in students*		16	5
Actions in the family sphere	*Actions involving parents/legal guardians/family*	Devices and their use within the family	12	4
		Training and information for parents/legal guardians/families	6	2
		Education and communication	19	12
		Other actions	6	2
	*Actions involving adolescents*		3	0
General actions	*Policy and legislation*		6	3
	*Science*		2	1
	*Other actions*		4	1
*Total actions*			134	52

### 3.3 Level of importance

In the third Delphi round, participants assessed the importance of each action in order to establish priorities. Seventeen experts took part in this round, corresponding to a retention rate of 77.27% relative to the second round.

Actions were rated on a 10-point scale. Based on the mean levels of importance, 54 actions (29.35%) were classified within the highest importance interval (9.0–10.0), 107 actions (58.15%) fell within the 7.0– < 9.0 range, 18 actions (9.78%) within the 5.0– < 7.0 range, and 5 actions (2.72%) within the 1.0– < 5.0 range (Table 5).

**Table 5:** Distribution of importance levels by action category.

Importance level interval	Total number of actions	Percentage of total actions	Number of actions by main category
1.0– < 5.0	5	2.72%	Educational sphere (*n =* 2)
			Family sphere (*n =* 3)
			General actions (*n =* 0)
5.0– < 7.0	18	9.78%	Educational sphere (*n =* 14)
			Family sphere (*n =* 3)
			General actions (*n =* 1)
7.0– < 9.0	107	58.15%	Educational sphere (*n =* 61)
			Family sphere (*n =* 34)
			General actions (*n =* 12)
9.0–10.0	54	29.35%	Educational sphere (*n =* 31)
			Family sphere (*n =* 20)
			General actions (*n =* 3)

At the thematic subsection level, ‘Teacher training' and ‘Science' achieved mean levels of importance above 9 (Supplementary Table S5). The highest number of actions with mean levels of importance equal to or greater than 9 was observed in the thematic subsection ‘Education and communication' within the family sphere, in ‘Classroom-based actions' within the educational sphere, and in the subcategory ‘Digital literacy and acquisition of psychosocial skills in students' (Supplementary Table S5).

### 3.4 Level of relevance

Overall, the main categories of actions in the educational sphere, actions in the family sphere, and general actions achieved mean agreement levels of 82.73%, 84.47%, and 86.11%, respectively, along with mean levels of importance of 8.24 (SD = 1.21), 8.24 (SD = 1.49), and 8.22 (SD = 0.79).

By integrating these two dimensions across the three Delphi rounds, actions were subsequently classified into three levels of relevance, reflecting the combined consideration of agreement and importance (Table 6). Level I (high relevance) included 38 actions (20.65%), Level II (moderate relevance) comprised 95 actions (51.63%), and Level III (limited relevance) included 51 actions (27.72%).

**Table 6:** Distribution of actions by level of relevance.

Category	Subcategory	Level I	Level II	Level III
Actions in the educational sphere	*Actions involving the educational center*	9	28	22
	*Actions involving teachers*	5	17	8
	*Digital literacy and acquisition of psychosocial skills in students*	5	11	3
Actions in the family sphere	*Actions involving parents/legal guardians/family*	16	27	13
	*Actions involving adolescents*	0	3	1
General actions	*Policy and legislation*	0	6	3
	*Science*	2	0	0
	*Other actions*	1	3	1
*Total actions*		*38*	*95*	*51*

Level I, high relevance; Level II, moderate relevance; Level III, limited relevance.

The distribution of actions by relevance level showed that actions involving parents, legal guardians, and/or families accounted for the highest number of Level I actions (*n* = 16; 8.70%), followed by actions involving the educational center (*n* = 9; 4.89%). The same subcategories also contributed the largest number of actions classified as Level II. Overall, actions within the educational and family spheres accounted for a greater number of Level I and Level II actions than general actions.

## 4 Discussion

The aim of this study was to reach expert consensus, using the Delphi technique, on actions to promote the healthy and safe use of ICT among adolescents. One of the most relevant findings is the convergence of expert perspectives from technical, health-related, and socio-educational backgrounds toward an approach centered on education and the family, moving beyond traditional paradigms based primarily on restriction and control.

This shift aligns with the findings of the EU Kids Online 2020 report, which identifies active mediation as the most desirable mediation strategy, as it fosters the development of digital skills and critical thinking. While restrictive mediation may reduce adolescents' exposure to certain risks, it may also limit opportunities for young people to develop the skills required to manage problematic situations independently (20).

The concentration of highly relevant actions within the subcategory ‘Actions involving parents/legal guardians/family' indicates that experts consider the home and family environment to be a primary context for support, safety, and learning in relation to healthy ICT use. In particular, the high ratings assigned to actions related to ‘Education and communication' suggests a clear prioritization of active mediation over restrictive approaches. Within the family sphere, this was reflected in the high levels of agreement and importance achieved by actions such as educating adolescents about the appropriate use of social media and the risks associated with inappropriate ICT use, with the aim of helping them identify harmful online behaviors [100% agreement; mean importance: 9.56 (SD = 1.03)], as well as promoting open dialogue with adolescents about the risks and benefits associated with ICT and Internet use [95.45% agreement; mean importance: 9.12 (SD = 1.22)]. In contrast, more restrictive approaches within the same sphere, such as prohibiting ICT use until the age of 14 inclusive, achieved substantially lower levels of agreement and importance [22.73% agreement; mean importance: 3.12 (SD = 2.20)]. This finding is consistent with previous research showing that restrictive mediation tends to be less effective during adolescence compared with other forms of parental mediation (21).

However, existing evidence also indicates that parents may experience difficulties in implementing well-focused active mediation, particularly when they perceive themselves as having limited digital literacy. This limitation may reduce the effectiveness of parental mediation in mitigating online risks (22). In this regard, the strong consensus observed in the thematic subsection ‘Training and information for parents/legal guardians/families' is especially relevant, as it underscores the need to equip families with the skills and resources necessary to promote healthy and safe technology use, in coordination with educational settings.

Within the educational sphere, actions associated with the subcategory ‘Teacher training' achieved mean levels of importance of 9 or higher, reflecting a perceived need for enhanced professional preparation to address health- and safety-related challenges linked to ICT use. This finding is consistent with previous studies indicating that a substantial proportion of teachers report low levels of confidence in identifying and responding to cyberbullying in educational contexts (23). In addition, research has highlighted the importance of adults serving as appropriate role models in relation to health-related information and behaviors on social media, particularly given that many adults continue to face difficulties in fully understanding these platforms and their dynamics (24).

Another key contribution of this study is the consensus achieved regarding actions within the subcategory ‘Digital literacy and acquisition of psychosocial skills in students', identified as essential components for promoting healthy and safe ICT use. These actions extend beyond purely technical considerations and are consistent with current evidence linking difficulties in emotional regulation to problematic internet use among adolescents (25). Moreover, higher levels of socio-emotional competence have been associated with a direct reduction in bullying and an indirect reduction in cyberbullying. Such competencies are also linked to a greater capacity to interpret, critically evaluate, and engage with digital content (26), which may contribute to a more protective profile against digital risks. Taken together, these findings support the integration of socio-emotional skills development and digital literacy as complementary strategies for risk mitigation.

The results also reveal a discrepancy between the overall volume of actions and their relative relevance. Although the educational sphere accounted for the largest number of actions identified, the subcategory ‘Actions involving parents/legal guardians/family' concentrated the highest number of highly relevant actions within the family sphere. This finding is noteworthy, as despite a similar number of actions being proposed for the educational center, family-related actions emerged as a strategic core within the overall set of recommendations.

Finally, the actions identified within the subcategory ‘Policy and legislation' are supported by the current European regulatory framework, particularly Regulation (EU) 2022/2065 (27), known as the Digital Services Act. The inclusion of actions beyond the educational and family spheres suggests that experts considered that interventions limited exclusively to schools and families may be insufficient to address the full range of risks associated with adolescents' ICT use. Accordingly, the actions proposed by the expert panel are aligned with Articles 34 and 35 of this Regulation, which apply to very large online platforms and very large online search engines. These services are defined as those with an average number of monthly active recipients in the European Union equal to or exceeding the threshold established in the Regulation and that have been formally designated as such by the European Commission.

Under this framework, designated services are required to assess and mitigate the systemic risks associated with the functioning and use of their platforms. In particular, the Regulation explicitly recognizes risks related to the protection of public health, the safeguarding of minors, and the prevention of serious negative consequences for individuals' physical and mental wellbeing, as well as other serious harms such as gender-based violence [Art. 34(1) (d)]. Beyond the obligation to conduct risk assessments, the Regulation also mandates the adoption of measures to reduce identified systemic risks, which may include, among others, specific measures aimed at protecting the rights of minors [Art. 35(1) (j)].

This regulatory alignment reinforces the notion that healthy and safe ICT use cannot rely exclusively on families and educational settings, but also entails binding legal obligations requiring digital services to adapt their design and operation in order to minimize harm. In this regard, the actions identified in the present study are consistent with the broader perspective that the protection of minors in digital environments should be understood as a shared responsibility involving families, educational settings, policymakers, and digital service providers. Strengthening industry-level protections may therefore contribute to a more balanced distribution of responsibilities for promoting safer and healthier digital environments among adolescents, alongside the preventive and educational roles undertaken by families, teachers, and schools.

Participant attrition across Delphi rounds represents one of the main limitations of this study, with the expert panel decreasing from 28 participants at baseline to 17 in the final round. Nevertheless, the lack of clear methodological guidance regarding optimal sample size in Delphi research suggests that numerical reduction alone should not be interpreted as a threat to validity (17). Accordingly, the study prioritized expert selection based on predefined inclusion criteria aimed at ensuring diversity in disciplinary backgrounds and professional experience, thereby enabling the integration of complementary perspectives and strengthening the robustness of the consensus obtained (14). In this sense, the panel brought together professionals from educational, health, university, and cybersecurity-related fields, although participants from secondary and vocational education represented the largest group.

A further methodological consideration concerns the volume of actions generated during the first round, which resulted in an extensive content framework for subsequent rounds. This complexity may have increased the time demands placed on panel members and, consequently, contributed to lower participation rates in later rounds. Despite this potential limitation, the decision was made to retain the full diversity and specificity of the contributions provided by the different professional profiles. This reflects a deliberate trade-off, prioritizing conceptual richness and analytical depth over procedural simplicity, in order to preserve the integrity and comprehensiveness of the proposed set of actions.

The aim of this Delphi study was to obtain multidisciplinary professional consensus from experts in education, health, and cybersecurity-related fields, consistent with the methodological principles of the Delphi technique. Future research could extend this expert consensus by conducting stakeholder validation with teachers, families, and adolescents. This sequential approach would allow the triangulation of expert consensus with lived experience, helping to identify potential gaps between expert-driven priorities and the perceived relevance and feasibility of the proposed actions among the populations directly affected. A next step would be to assess each relevant action using brief measures of perceived relevance and feasibility, complemented by qualitative feedback, to refine the framework and support its subsequent dissemination and implementation. Future research could also explore whether some actions are particularly relevant for younger or older adolescents, as well as identify actions that may be applicable across different developmental stages of adolescence.

Within this context, the consensus reached points to a shift in focus in adolescent digital health and safety, positioning education and active mediation as central strategies over predominantly restrictive approaches.

A comprehensive analysis of the findings indicates that, while the educational sphere generated the largest volume of actions, the family sphere emerged as a priority area for intervention. Notably, one of its subcategories concentrated the highest number of highly relevant actions across all subcategories analyzed. However, the results suggest that the effectiveness of this support depends both on adolescents' digital literacy and the development of psychosocial skills, as well as on equipping families and teachers with adequate training in digital competencies and the promotion of emotional health. Differences in families' levels of digital literacy and their capacity to provide active mediation may further influence the feasibility of some proposed actions across contexts. Future research should therefore explore these contextual differences, as well as potential strategies to reduce inequalities in the implementation of digital health and safety initiatives. In addition, within this framework, actions aligned with current European regulations were identified, alongside the need to extend their implementation to other legislative contexts.

Overall, the actions agreed upon by the panel provide a solid foundation for the design of policies and programmes aimed at fostering a healthy digital environment, where the protection of adolescents is based on competence, autonomy, and shared responsibility, rather than on control-based measures alone. In this context, these findings offer an evidence-informed and prioritized starting point for stakeholder validation and the development of scalable strategies to promote adolescent digital health and safety.

## Data Availability

The datasets generated and/or analyzed during the current study are available from the corresponding author on reasonable request, with any data shared being fully anonymized to maintain participant confidentiality and privacy related to the Delphi process.
