# Supplementary material for: Consensus-based framework of actions within educational and family settings to promote healthy and safe ICT use among adolescents: a Delphi study
Source: Front Public Health. 2026 Jun 24;14:1813279. doi: 10.3389/fpubh.2026.1813279 (PMC13341724; doi:10.3389/fpubh.2026.1813279)
Supplement: Supplementary file 1 [file Data_Sheet_1.docx]

Supplementary Material

[*1.* Table S1. *Sociodemographic characteristics of the expert panel (Round 1)* 3](#_Toc229128080)

[2. Delphi questionnaire S2 4](#_Toc229128081)

[*3.* Table S3. *Categories, subcategories, thematic subsections, and illustrative examples derived from the proposed actions.* 5](#_Toc229128082)

[*4.* Table S4. *Mean levels of agreement and importance across thematic subsections.* 8](#_Toc229128083)

[*5.* Table S5. *Actions with a mean importance score ≥ 9: distribution, number, and percentage.* 9](#_Toc229128084)

## Table S1. *Sociodemographic characteristics of the expert panel (Round 1)*

| **Variable** | **n (%)** |
| --- | --- |
| ***Sex*** |  |
| Female | 18 (64.29%) |
| Male | 10 (35.71%) |
| ***Geographical location*** |  |
| Spain | 26 (92.86%) |
| Andalusia | 20 (71.43%) |
| Other regions of Spain | 6 (21.43%) |
| France | 1 (3.57%) |
| Colombia | 1 (3.57%) |
| **Total** | **28 (100%)** |

*Percentages are calculated based on the total sample (n = 28).*

## Delphi questionnaire S2

**Round 1. Generation of actions**

*Operational definition of safe ICT use*

We understand this as the responsible and balanced use of the internet, social media, and video games through devices such as smartphones, tablets, or computers, avoiding excessive use that may lead to addiction. It includes protection against exposure to inappropriate content, such as toxic health-related habits, violence, or pornography, as well as protection against online sexual solicitation. It also encompasses the prevention of harassment or violent behaviours, the protection of privacy, and the prevention of personal content leakage. Overall, it promotes a healthy and safe digital environment, fostering adolescents’ emotional and physical wellbeing.

*Open-ended question*

What actions can be implemented in educational and family settings to ensure that adolescents make safe use of ICTs and thereby prevent associated risks and social media addiction?

*Response format*

Open-ended free-text response.

**Round 2. Level of agreement**

In the second round, participants were asked to review the list of actions generated in Round 1 and to indicate their level of agreement with each proposed action.

*Response format*

- Agree
- Disagree

**Round 3. Level of importance**

In the third round, participants were asked to rate the importance of each action.

*Response format*

Importance was rated on a 10-point scale, ranging from 1 (lowest importance) to 10 (highest importance).

*Controlled feedback*

Information on the level of agreement reached in the previous round was provided as controlled feedback to support informed judgement.

## Table S3. *Categories, subcategories, thematic subsections, and illustrative examples derived from the proposed actions.*

| **Category** | **Subcategory** | **Thematic subsection** | **Illustrative examples derived from the proposed actions** |
| --- | --- | --- | --- |
| **Actions in the educational sphere** | *Actions involving the educational centre* | Creation and implementation of protocols and regulations for ICT device security and use | *Activating protocols to prevent and respond to cyberbullying episodes.* |
|  |  | Resource mobilisation and development of digital literacy programmes | *Requesting official programmes on safe ICT use, cyberbullying, social media addiction, and other online risks.* |
|  |  | Actions involving families and other specialists | *Collaboration between educational and family settings through joint workshops on safe ICT use, coordinated rules between home and school, and practical activities such as digital use agreements or screen-free events.* |
|  |  | Other actions | *Early detection of warning signs and risk behaviours such as excessive online time, social isolation, or social media-related anxiety.* |
|  | *Actions involving teachers* | Teacher training | *Teacher training on responsible ICT use, online risks and addictions, evidence-based school prevention programmes, and strategies to promote respectful and healthy online behaviours, communication skills, and emotional well-being among adolescents.* |
|  |  | Classroom-based actions | *Incorporating digital education by teaching responsible ICT use, the consequences of misuse, and skills to protect digital identity.* |
|  | *Digital literacy and acquisition of psychosocial skills in students* |  | *Implementing preventive and socio-emotional education programmes that address individual and group risk and protective factors and provide evidence-based tools to cope with digital challenges.* |

**Table S3.** *Categories, subcategories, thematic subsections, and illustrative examples derived from the proposed actions* (*Continued*).

| **Category** | **Subcategory** | **Thematic subsection** | **Illustrative examples derived from the proposed actions** |
| --- | --- | --- | --- |
| **Actions in the family sphere** | *Actions involving parents/legal guardians/family* | Devices and their use within the family | *Establishing clear and agreed family rules through dialogue with adolescents, including limits on screen time, schedules, and device use for both educational and recreational purposes.* |
|  |  | Training and information for parents/legal guardians/families | *Evidence-based family prevention programmes delivered by trained professionals to promote responsible and positive ICT use and prevent online risks and addictive behaviours through parental training on digital literacy, online risks, positive digital mediation, communication skills, healthy technology habits, and the promotion of adolescents’ emotional well-being and healthy leisure activities* |
|  |  | Education and communication | *Adults should serve as role models and lead by example in the responsible use of technology by promoting healthy device use, digital disconnection, critical consumption of online content, and the protection of personal information* |
|  |  | Other actions | *Seeking support from specialised professionals when digital-related problems or early warning signs are detected.* |
|  | *Actions involving adolescents* |  | *Teaching adolescents about their rights and responsibilities regarding online content sharing, privacy, consent, and the consequences of sharing images or personal information on social media* |

**Table S3.** *Categories, subcategories, thematic subsections, and illustrative examples derived from the proposed actions* (*Continued*).

| **Category** | **Subcategory** | **Thematic subsection** | **Illustrative examples derived from the proposed actions** |
| --- | --- | --- | --- |
| **General actions** | *Policy and legislation* |  | *Implementing legislative measures to protect minors by prioritising ethical and educational considerations over commercial interests in digital environments.* |
|  | *Science* |  | *Implementing evidence-based interventions to promote responsible ICT use and prevent or reduce associated risks among adolescents.* |
|  | *Other actions* |  | *Integrating multidisciplinary and cross-sector efforts to create a holistic approach to the prevention of ICT-related risks among adolescents.* |

## Table S4. *Mean levels of agreement and importance across thematic subsections.*

| **Category** | **Subcategory** | **Thematic subsection** | **Mean level of agreement (%)** | **Mean level of importance (SD)** |
| --- | --- | --- | --- | --- |
| **Actions in the educational sphere** | *Actions involving the educational centre* | Creation and implementation of protocols and regulations for ICT device security and use | 75.55% | 7.83 (1.41) |
|  |  | Resource mobilisation and development of digital literacy programmes | 92.04% | 8.93 (0.59) |
|  |  | Actions involving families and other specialists | 89.06% | 8.80 (0.58) |
|  |  | Other actions | 77.27% | 7.61 (0.99) |
|  | \|  \| \| --- \|  \| *Actions involving teachers* \| \| --- \| | Teacher training | 89.39% | 9.25 (0.24) |
|  |  | Classroom-based actions | 83.33% | 8.15 (1.41) |
|  | *Digital literacy and acquisition of psychosocial skills in students* |  | 86.12% | 8.43 (0.91) |
| **Actions in the family sphere** | *Actions involving parents/legal guardians/family* | Devices and their use within the family | 76.14% | 7.63 (2.22) |
|  |  | Training and information for parents/legal guardians/families | 87.88% | 8.51 (0.55) |
|  |  | Education and communication | 92.27% | 8.89 (0.51) |
|  |  | Other actions | 83.64% | 8.01 (1.07) |
|  | *Actions involving adolescents* |  | 84.09% | 8.23 (1.03) |
| **General actions** | \|  \| \| --- \|  \| *Policy and legislation* \| \| --- \| |  | 85.41% | 8.07 (0.71) |
|  | *Science* |  | 95.46% | 9.12 (0.17) |
|  | *Other actions* |  | 83.64% | - 1. (0.92) |

| **Category** | **Subcategory** | **Thematic subsection** | **Actions** ≥ **9**  **n (%)** |
| --- | --- | --- | --- |
| **Actions in the educational sphere** | *Actions involving the educational centre* | Creation and implementation of protocols and regulations for ICT device security and use | 5 (2.72%) |
|  |  | Resource mobilisation and development of digital literacy programmes | 4 (2.17%) |
|  |  | Actions involving families and other specialists | 5 (2.72%) |
|  |  | Other actions | 1 (0.54%) |
|  | *Actions involving teachers* | Teacher training | 3 (1.63%) |
|  |  | Classroom-based actions | 7 (3.80%) |
|  | \|  \| \| --- \|  \| *Digital literacy and acquisition of psychosocial skills in students* \| \| --- \| |  | 6 (3.26%) |
| **Actions in the family sphere** | *Actions involving parents/legal guardians/family* | Devices and their use within the family | 5 (2.72%) |
|  |  | Training and information for parents/legal guardians/families | 1 (0.54%) |
|  |  | Education and communication | 10 (5.43%) |
|  |  | Other actions | 3 (1.63%) |
|  | \|  \| \| --- \|  \| *Actions involving adolescents* \| \| --- \| |  | 1 (0.54%) |
| **General actions** | *Policy and legislation* |  | 0 (0%) |
|  | *Science* |  | 2 (1.09%) |
|  | *Other actions* |  | 1 (0.54%) |
| ***Total actions*** |  |  | 54 (29.35%) |

## Table S5. *Actions with a mean importance score ≥ 9: distribution, number, and percentage.*
